# Effects of phenytoin injection on vocal cord healing after mechanical trauma: An experimental study

**DOI:** 10.3906/sag-1903-63

**Published:** 2019-10-24

**Authors:** Sibel ALİCURA TOKGÖZ, Cem SAKA, İstemihan AKIN, Fulya KÖYBAŞIOĞLU, Saffet KILIÇASLAN, Murat ÇALIŞKAN, Ömer BEŞALTI, Emel ÇADALLI TATAR

**Affiliations:** 1 Department of Otorhinolaryngology, University of Health Sciences Dışkapı Yıldırım Beyazıt Training and Research Hospital, Ankara Turkey; 2 Department of Pathology, Faculty of Medicine, Yüksek İhtisas University, Ankara Turkey; 3 Department of Otorhinolaryngology, Düzce Atatürk State Hospital, Düzce Turkey; 4 Department of Surgery, Faculty of Veterinary Medicine, Ankara University, Ankara Turkey

**Keywords:** Phenytoin, vocal cord, wound healing, rat

## Abstract

**Background/aim:**

Phenytoin is an anticonvulsant drug which causes fibroblast proliferation, collagen synthesis, and an increase in epidermal growth factor. Therefore, the aim of the present study is to evaluate the effect of phenytoin injection on the wound healing process in rats with vocal cord injury by histopathological methods.

**Materials and methods:**

The vocal cords of 10 albino Wistar rats were damaged bilaterally; the left vocal cord was kept as the control group. Phenytoin was injected in the right vocal cord. Ten rats were sacrificed. The thickness of the lamina propria and density of the fibroblast and collagen were evaluated histopathologically.

**Results:**

Thickness of the lamina propria was 18.0 ± 7.1 µm in the control group, 65.5 ± 10.7 µm in the phenytoin group. The density of fibroblast and collagen were statistically lower in the control group compared the phenytoin group (P < 0.05).

**Conclusion:**

Phenytoin injection in rats after vocal cord injury significantly increased the thickness of the lamina propria and density of fibroblast and regular and mature collagen in the lamina propria. The findings in our study provide a feasible scientific view for adding phenytoin treatment to vocal cord surgeries in otolaryngology practice, but further studies are needed in order to evaluate the use of phenytoin in preventing the formation of scar tissue and possible effects on vocal cord vibration in humans after vocal cord injury.

## 1. Introduction

Vocal cord injury is a significant problem which affects quality of life and causes severe voice disturbances [1]. Scar tissue usually develops after surgery, radiotherapy, and inflammation [2]. This leads to decreased viscoelasticity of the vocal fold lamina propria (increased stiffness), which reduces the vibration potential of the vocal folds [3]. The voice is often breathy or aphonic and the phonation threshold pressure is elevated [4].

There are no recent effective treatments for preventing vocal cord injury. The treatment is usually difficult and may include voice therapy with a speech and language therapist and injection augmentation. Many substances have been tried. In order to discover an effective treatment modality, events occurring in the early stages after vocal cord injury should be known. A fibrin coat is seen in the injury site on the first day; the fibrin coat gives way to cellular aggregation on the third day of the wound healing process after trauma. New collagen production occurs on the fifth day, and mature collagen aggregation occurs on the seventh day [5,6].

Agents like autologous fat, hepatocyte growth factor, fascia, collagen, hyaluronic acid, and hydroxyapatite and platelet-rich plasma have been used to treat vocal cord injury in literature. However, the efficacy of these treatments is usually limited; hence, these treatments are unable to recover the normal distribution of extracellular matrix components [7–10].

Phenytoin (diphenylhydantoin sodium – DpH) is an anticonvulsant drug used in grand mal and psychomotor epilepsy; its positive effects on wound healing have been mentioned in recent publications. In a review, it was found that phenytoin causes fibroblast proliferation, collagen synthesis, and increase in epidermal growth factor with the same mechanism causing gingival hypertrophy as a side effect of systemic phenytoin use; it induces new vessel formation, speeds the decrease of microbial colonies, reduces pain and inflammation, and improves healing [11]. In various studies, it has been found that topical phenytoin has important positive effects in the recovery period for skin ulcerations, burns, and diabetic wounds [12]. Furthermore, it has been reported that phenytoin decreases wound contraction and development of scar tissue [13].

In this study, we aimed to evaluate the effects of phenytoin injection in the recovery of vocal cord injury in rats.

## 2. Materials and methods

### 2.1. Study design

Ethical approval for animal experimentation was obtained from Ankara University’s Animal Experimentation Ethics Committee with approval number 2011-119-461. Ten adult male healthy albino Wistar rats weighing 250–270 grams from Hıfzısıhha Laboratory were used. Rats were kept in appropriate temperature and humidity conditions for 48 h in order to adapt after transportation. Rats were kept at Ankara University Faculty of Veterinary’s Surgery Clinic in an environment with humidity of 65%–70% and temperature of 22 ± 2 °C in 12 h of light and 12 h of dark, with free food and water supply. Every clause in the Helsinki Final Act applicable to experimental studies was complied with. 

### 2.2. Administration of drug and anesthetics

**Figure 1:**
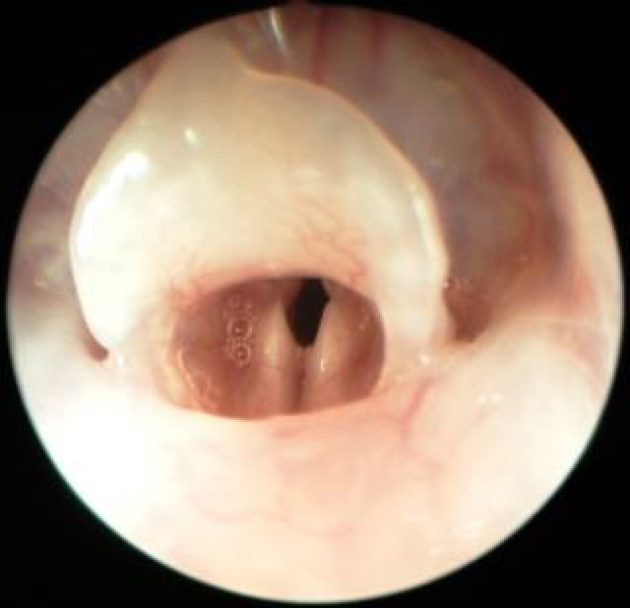
Superior view of the rat larynx through the endoscope

**Figure 2:**
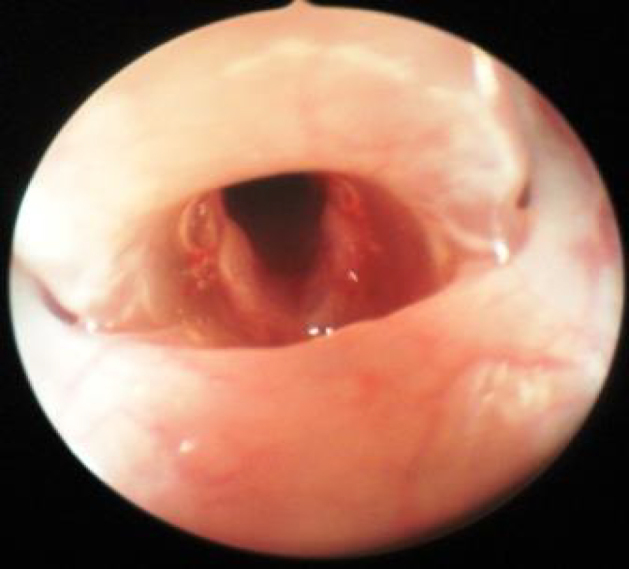
A wound surgically created on the bilateral vocal cord.

Each rat received 60 mg/kg intraperitoneal (i.p.) ketamine hydrochloride (Ketalar®, Eczacıbaşı Parke-Davis, İstanbul, Türkiye) and 10 mg/kg i.p. Xylazine HCl (Alfazyne®, Alfasan International B.V. Woerden, The Netherlands). Rectal temperature was measured regularly while the rats were under anesthesia, and they were placed onto warm blankets in order to keep their body temperature at 35 °C. Endoscopic direct laryngoscopy was performed with a 30° 2.7-mm pediatric telescope; the right vocal cords were considered the phenytoin group and left vocal cords the control group (Figure 1.) The lesions were made with Richards 1-mm cup forceps by the same surgeon on the 1/3 front, 2/3 back meeting point of both vocal cords (Figure 2). Phenytoin was then administered with 0.05 mL ppd injection lateral of the lesion made to the upper side of the right vocal cord. The rats were sacrificed for histopathological examination 15 days later. 

### 2.3. Histopathological examination

**Figure 3:**
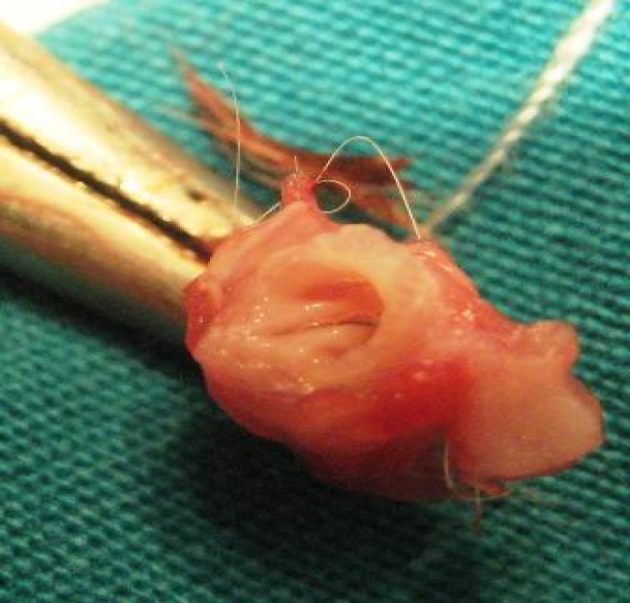
The view of the larynx and vocal cords after dissection.

Histopathological evaluation was performed by pathologists who were blinded for study groups. Vocal cords were removed by dissection while preserving their integrity (Figure 3). After fixation in 10% formaldehyde solution, the vocal cords were decalcified in 10% formic acid solution for 24 h. Macroscopic sampling was performed with the supervisor. The material was processed as a whole in order to evaluate the vocal cords. After routine paraffin processing, the parameters mentioned below were evaluated in hematoxylin–eosin (H & E) and Masson–Trichrome staining with an Olympus BX 50® light microscope using 5-mm–thick sections. The density of collagen and fibroblast in the lamina propria was measured by dividing each vocal cord cross-section into 20 unit areas, and calculated as a percentage.

### 2.4. Statistical analysis

Data were analyzed with the SPSS for Windows 11.5 package program (SPSS, Inc., Chicago, IL, USA). The Shapiro–Wilk test was used to determine whether the distribution of continuous variables was close to normal. Descriptive statistics were shown as mean ± standard deviation or median (minimum–maximum) for continuous variables. The difference of the mean thickness of the lamina propria between the control and study groups was evaluated with the dependent t-test, and the difference of median values ​​of collagen and fibroblast densities was evaluated by the Wilcoxon Sign test. Results with P < 0.05 were considered statistically significant.

## 3. Results

**Figure 4:**
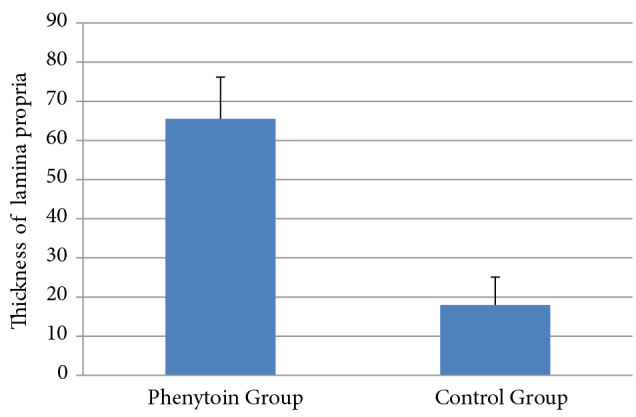
Comparison of thickness of the lamina propria by group.

**Figure 5:**
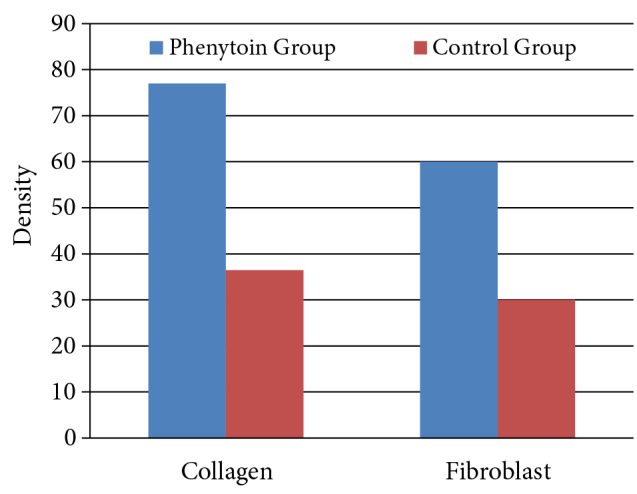
Comparison of collagen and fibroblast density by group.

Thickness of the lamina propria was 18.0 ± 7.1 µm in the control group, 65.5 ± 10.7 µm in the phenytoin group. The thickness of the lamina propria was significantly higher in the phenytoin group than in the control group (P < 0.001; Figure 4). Density of collagen was 36.5% (25.0–40.0) in the control group, 77.0% (72.0–83.0) in the phenytoin group, and density of fibroblast was 30.0% (25.0–35.0) in the control group, 60.0% (60.0 –70.0) in the phenytoin group. There was a statistically significant difference between the groups for both collagen and fibroblast density, and the intensity levels in the control group were found to be lower than in the phenytoin group (P = 0.005; Figure 5; Table).

**Table:** Lamina propria thickness and collagen and fibroblast density in control and phenytoin groups.

Variables	Control group	Phenytoin group	P-value
Thickness of lamina propria (µm)	18.0 ± 7.1	65.5 ± 10.7	<0.001
Density of collagen (%)	36.5(25.0–40.0)	77.0(72.0–83.0)	0.005
Density of fibroblast (%)	30.0(25.0–35.0)	60.0(60.0–70.0)	0.005

## 4. Discussion

In adults, vocal cords have a structure of three layers: epithelium, lamina propria (superficial, intermediate, and deep layers), and musculus vocalis. The superficial layer of the lamina propria and epithelium are essential for vibration and phonation. Surgical trauma affects these two layers in particular. Acute response after injury is vital in the healing process. The proliferative phase starts 2–3 days after injury and ends after 2–3 weeks [8]. Interfering with the formation of neomatrix by altering the cell phenotype can change the functional results for the damaged vocal cord.

Different animal models have been used in studies of vocal cord scarring. Rats have advantages over other models; it has been shown that the rat vocal cord’s lamina propria has similar characteristics with the human vocal cord’s lamina propria. The lamina propria of rats is divided into three layers; the deep layer contains more collagen fibers than the superficial layer [14]. 

Various agents have been used to accelerate wound healing after vocal cord injury. Yıldız et al. used estradiol and dexamethasone in the rat model with vocal cord injury. They did not find any significant differences when comparing the treated group with the group receiving saline [15]. In another study, basic fibroblast growth factor was shown to reduce vocal cord scar formation [16]. Hirano et al. found increased viscoelastic properties and less scar tissue formation after Hepatocyte Growth Factor injection [8].

Phenytoin accelerates wound healing with fibroblast proliferation, collagen deposition, collagenase enzyme inhibition, and antibacterial activity pathways. Fibroblast proliferation, the production of the extracellular matrix and its proteins, and the activity of growth factors and their mediators may be increased within the wound. Ultimately, collagen production and deposition are increased, resulting in enhanced wound strength [12,17]. Furthermore, the effects of neural membrane stabilization and local inflammatory reaction limitation are other useful properties demonstrated in the literature [11,18]. Acceleration of wound healing after surgical treatment in vocal cord pathologies is a direct contributor to success and patient satisfaction. In cases where wound healing is poor, negative functional results may be seen even after a successful surgery. Thus, the effects of phenytoin inducing fibroblast proliferation and collagen deposition may play an essential role in the treatment of surgical vocal cord injury. The efficacy, safety, ease of use, and low cost of phenytoin as an antiepileptic drug suggests the possibility that it can also be used prophylactically in adjuvant therapy after nasal surgery [19]. In various studies and reviews, it has been reported that phenytoin can be used topically for wounds in skin and soft tissues and various types of ulcers [17,20,21].

In a study by Tateya et al. [14] in rats with vocal cord injury, collagen type I peaks 2 weeks after lesion formation and then gradually decreases. Similarly, fibronectin density synthesized by fibroblasts peaks and decreases gradually after 2 weeks. In our study, in the phenytoin group, fibroblast and collagen density increased, but collagen fibers were more regular and mature than the control group. Phenytoin has also been reported to enhance the maturation of collagen in both normal skin and granulation tissue in rats, possibly by promoting collagen crosslinking [22]. 

Previous studies have shown that the thickness of the lamina propria is higher in the scarred rabbit vocal cord than the normal vocal cord [23,24]. In our study, in the phenytoin group, thickness of the lamina propria was significantly greater than in the control group. Although phenytoin has antiinflammatory properties, the reason for the pronounced thickness of the lamina propria in the phenytoin group is increased collagen density. There has been no previous study in the literature that showed the effects of phenytoin on vocal cord lamina propria. In our study, rats were sacrificed on the 15th day for histopathological evaluation. The thickness of the lamina propria can be measured for a longer follow-up for the evaluation of the effects of phenytoin. 

Surgical treatment is the primary treatment option in vocal cord diseases. In order to recover the patient’s voice quickly and with good quality, and to preserve the mucosal wave, diligence is essential. The best contribution of this study to the literature is that it is the first study to evaluate phenytoin injection in vocal cord injury. However, the effects of phenytoin on vocal cord vibration was not determined; hence, an experimental model showing vocal cord functions could not be established.

This experimental study has several limitations, such as the small group sizes. The small size issue occurred due to our ethical concern regarding the “principle of reduction” in animal experiments; however, larger numbers would be needed to reach a clear conclusion on the topic. The second limitation is that we used a subjective scoring system to evaluate the histopathologic changes in the vocal cord samples. It would be more accurate to use an image analysis program that allows for objective/automated interpretation of the healing of scarred vocal cords and histopathological changes, which will facilitate a clear conclusion.

It has been shown in this study that phenytoin, which improves wound healing processes in various tissues, has positive effects in rat vocal cords. It is relatively easy to handle and inject, although as with all other materials, there is variability to the final location of the injectate. New collagen and fibroblast synthesis is stimulated in response to injection of phenytoin. However, the phenytoin was not shown to improve the biomechanical properties of the scarred vocal fold. According to these findings, phenytoin may be more appropriately used to medialize a stiffened scarred vocal fold that is contributing to glottic insufficiency rather than to restore optimal viscoelastic properties of a deficient lamina propria. The findings in our study provide a feasible scientific view for adding phenytoin treatment to vocal cord surgeries in otolaryngology practice. The rapid improvement of the patient’s voice quality may increase patient satisfaction and improve the results of the operation. Other experimental and clinical studies should support the data we obtained.
